# Community-Based Health and Exposure Study around Urban Oil Developments in South Los Angeles

**DOI:** 10.3390/ijerph15010138

**Published:** 2018-01-15

**Authors:** Bhavna Shamasunder, Ashley Collier-Oxandale, Jessica Blickley, James Sadd, Marissa Chan, Sandy Navarro, Michael Hannigan, Nicole J. Wong

**Affiliations:** 1 Urban and Environmental Policy Department, Occidental College, Los Angeles, CA 90041-3314, USA; marissawchan@gmail.com; 2 Department of Environmental Engineering, University of Colorado Boulder, Boulder, CO 80309-0427, USA; Ashley.Collier@Colorado.edu; 3 Center for Digital Liberal Arts, Occidental College, Los Angeles, CA 90041-3314, USA; blickley@oxy.edu; 4 Geology Department, Occidental College, Los Angeles, CA 90041-3314, USA; jsadd@oxy.edu; 5 Esperanza Community Housing, Los Angeles, CA 90007, USA; snavarro213@gmail.com; 6 Department of Mechanical Engineering, University of Colorado Boulder, Boulder, CO 80309-0427, USA; hannigan@colorado.edu; 7 Redeemer Community Partnership, Los Angeles, CA 90018, USA; niki@redeemercp.org

**Keywords:** oil and gas development, urban oil drilling, cumulative impacts, environmental justice, community-based participatory research, health survey, low-cost sensors, methane

## Abstract

Oilfield-adjacent communities often report symptoms such as headaches and/or asthma. Yet, little data exists on health experiences and exposures in urban environments with oil and gas development. In partnership with *Promotoras de Salud* (community health workers), we gathered household surveys nearby two oil production sites in Los Angeles. We tested the capacity of low-cost sensors for localized exposure estimates. Bilingual surveys of 205 randomly sampled residences were collected within two 1500 ft. buffer areas (West Adams and University Park) surrounding oil development sites. We used a one-sample proportion test, comparing overall rates from the California Health Interview Survey (CHIS) of Service Planning Area 6 (SPA6) and Los Angeles County for variables of interest such as asthma. Field calibrated low-cost sensors recorded methane emissions. Physician diagnosed asthma rates were reported to be higher within both buffers than in SPA6 or LA County. Asthma prevalence in West Adams but not University Park was significantly higher than in Los Angeles County. Respondents with diagnosed asthma reported rates of emergency room visits in the previous 12 months similar to SPA6. 45% of respondents were unaware of oil development; 63% of residents would not know how to contact local regulatory authorities. Residents often seek information about their health and site-related activities. Low-cost sensors may be useful in highlighting differences between sites or recording larger emission events and can provide localized data alongside resident-reported symptoms. Regulatory officials should help clarify information to the community on methods for reporting health symptoms. Our community-based participatory research (CBPR) partnership supports efforts to answer community questions as residents seek a safety buffer between sensitive land uses and active oil development.

## 1. Introduction

The consequences for public health of oil development in densely populated urban environments is an understudied research question, but one of great concern to residents surrounding oil and gas development facilities in Los Angeles. Oil and natural gas extraction activity, both using traditional primary production methods as well as secondary methods such as flooding, steam injection, hydraulic fracturing, and acidization, has prompted concerns over impacts to water, air, land, and public health across the United States [1]. Yet, there is little environmental or public health data on the consequences of oil development in urban core cities, such as Los Angeles, with substantial production located near dense populations. The National Institute of Environmental Health Sciences funded Environmental Health Sciences Core Centers recommended that future research should examine exposure and health outcomes related to oil and natural gas development with community engagement as central to study design [2]. Community-based participatory research (CBPR) can be a vital component of research that is designed around the 3Rs—rigor, reach, and relevance—that is critical to the usefulness of scientific findings [3]. Our study is a contribution to CBPR research in an urban oil development context. We examined oil development in South Los Angeles through self-reported community health. We also gathered qualitative information about community knowledge of and experiences living nearby an oil development site. Finally, we tested a low-cost method for identifying methane emissions from oil development in a neighborhood impacted by multiple pollution sources to better discern oil-related exposures. 

Research studies on the health impacts of proximity to oil and gas development suggest an important spatial dimension, with those living closer to active wells experiencing greater adverse impacts. Residents living within 0.8 km from a gas well, compared with residents living further away from such active gas development, experience greater impacts on their health from exposure to gas emissions [4]. Greater density and proximity of natural gas wells to maternal residence (within a 10 m radius) were associated with adverse birth outcomes [5]. Residential proximity has also been associated with dermal and respiratory conditions in residents near natural gas extraction activities with distances typically measured at less than a kilometer to two kilometers from well to residence [6]. 

Los Angeles is an oil-rich basin concentrated across 70 active oil fields [7]. In some neighborhoods, residences are located just 3 ft. away from the boundary of an active drilling and/or production site, with the wellheads as close as 60 ft. from the residence. In Los Angeles County, a population of 10 million people resides amidst more than 5000 active oil and gas wells, with the City of Los Angeles hosting approximately 850 active wells and 4750 wells in the county, adjacent to over 4 million and 10 million residents, respectively. In neighborhoods such as South Los Angeles, wells are located nearby a dense residential population and sensitive land uses such as childcare centers, schools, urban parks and playgrounds, and senior residential and healthcare facilities, many composed of vulnerable populations [8,9]. The proximity of oil wells and production facilities to a dense population makes Los Angeles a critical site for examining the public health consequences and potential exposures from oil and gas development in an urban context. Our community-based health and exposure study is a collaboration between Occidental College, University of Colorado, Boulder, and member organizations of STAND-LA (Stand Together against Neighborhood Drilling-Los Angeles). STAND-LA is a coalition of environmental justice and community groups organized to confront the public health consequences of oil development for residents living amidst active oil development in the City of Los Angeles. Despite the more than 100-year history of oil development in Los Angeles [10], there is a dearth of data on environmental and public health consequences of these long-lived and continuous operations. We sought to generate baseline community data by conducting a preliminary survey through the collection of comparable self-reported health data as well as baseline information about resident experiences of living near active oil development. Further, communities have no access to exposure monitoring data, and air monitoring can be prohibitively expensive [11]. Low-cost sensors are a potential option for generating localized community-relevant monitoring data that can be examined alongside self-reported data. 

Los Angeles is a well-documented “riskscape” of environmental hazards and elevated health risk, disproportionately concentrated in poor communities and communities of color [12,13]. CalEnviroscreen, a pollution exposure and health vulnerability screening tool developed by the California Environmental Protection Agency Office of Environmental Health Hazard Assessment for use by State agencies, helps identify communities most affected by multiple sources of pollution, and where people are especially vulnerable to pollution’s effects [14]. CalEnviroscreen calculates scores that reflect cumulative impact and vulnerability for every census tract in the state using environmental, health, and socioeconomic information from state and federal government regulatory agencies (Cushing et al. 2015). Vulnerability incorporates individual and community level characteristics that make people more sensitive to pollution’s effects, such as young children and people with asthma, and socioeconomic factors, such as poverty, race/ethnicity, and education. The tool ranks communities based on their scores, and then maps these scores, allowing for objective cross-community comparisons. Because the determinants of vulnerability include socioeconomic factors, such as race/ethnicity and income, our CBPR study examines active oil production in two communities with a residential population that is poorer, and with a higher proportion of non-Anglo people than the city or county overall. The West Adams and University Park neighborhoods, surrounding the Jefferson (The Jefferson Drill Site) and AllenCo (the AllenCo Drill Site) oil production sites, are both located in an area identified by CalEnviroscreen as an “environmental justice” community, defined as among the top 25% of most environmentally most environmentally burdened census tracts in the state [15]. 

Environmental justice (EJ) community organizations have historically recognized and documented health and environmental consequences from polluting industries and other incompatible land uses located near their homes, schools, and playgrounds [16,17]. EJ communities often more actively seek information and gather data related to community hazards to demonstrate the hazards and risks they face, and to better inform policy and decision-making [18]. Residents in Los Angeles neighborhoods near oil development sites report health symptoms such as nosebleeds and headaches, ailments that have also been described with oil and gas production in other areas [19]. Oil production and drilling is associated with exposure to hazardous air pollutants (HAPs) and toxicants, such as BTEX chemicals (benzene, toluene, ethylbenzene, and xylene) [20]. 

Secondary drilling and production enhancement practices inject fluids into oil and gas reservoir rocks to enhance the recovery of the hydrocarbon products. For example, acidizing is used in Los Angeles, where large volumes and high concentrations of hydrochloric acid, hydrofluoric acid, or other chemicals are injected underground, mixing and reacting with other well fluids, most of which lack adequate hazard evaluation. Oil development facilities within the South Coast Basin submit chemical use reports for certain well activities. The reports indicate chemical ingredients with known air toxics such as hydrogen chloride, xylene, hydrofluoric acid, and ethylbenzene used as part of standard well development and maintenance acidizing practices [21,22]. 

Residents living near active oil wells and production facilities in Los Angeles often note symptoms such as nosebleeds, headaches, and worsened asthma. In the most highly publicized case, residents of the University Park neighborhood in 2013 complained of foul emissions and reported nosebleeds, headaches, and respiratory problems. These complaints coincided with increased oil production in the nearby field, where production rose 400% in one year following the purchase of the facility by AllenCo Energy Inc. (Los Angeles, CA, USA) (4178 barrels in 2009 to 21,239 barrels in 2010 [23]. Subsequently, the EPA fined AllenCo. The facility is temporarily closed with plans to reopen once it installs emissions control equipment. Other extraction facilities and wells in this and many other densely populated Los Angeles neighborhoods continue to operate. 

As proximity to oil and gas development has emerged as important to understanding its public health impacts, policy measures suggest the need for setbacks or buffers as a public health protection [24,25]. Los Angeles requires no buffers or setbacks from oil development operations, which permits very close distances between residents and extraction sites. Responding to community complaints, in April 2017, Los Angeles City Council introduced a motion for the city to study the possibility of a safety buffer [26]. 70% of active wells in Los Angeles are located within a 1500 ft. distance from “sensitive land uses” [27], such as a home, school, childcare facility, urban park or playground, or senior residential facility, as defined by Cal EPA (Table 1). Setbacks have been enacted in municipalities in Colorado, Pennsylvania, and Texas to separate oil and gas development from residences for health and safety protections. Here, we report on the analysis of a random sample household survey of residents living within a 1500 ft. radius of oil development sites. We compare resident self-reported health within the 1500 ft. radius of the site to resident health in Service Planning Area 6 (SPA6), the Los Angeles County Department of Public Health designated area in which South Los Angeles is located and to Los Angeles County residents overall. 

In partnership with residents, we also piloted the use of an open-source low-cost air quality monitoring system during the survey period in West Adams (Jefferson Oil Field) as a pilot site. While these sensors present challenges in terms of cross-sensitives and lower accuracy/precision compared with conventional monitoring equipment [28], they have led to more accessible tools that can complement existing monitoring methods and serve as a screening method for concerned communities. In recent years, much work has gone into understanding the capabilities of low-cost sensors [29,30,31] and they have been utilized in a variety of applications from personal exposure monitoring [32] to high-density networks designed for monitoring in complex urban environments [33]. Sensors have even been calibrated for the detection of ambient levels of methane [34]. We present preliminary results from the calibration and deployment of these same sensors in areas with and without oil and gas activity and discuss how this type of data may further support CBPR efforts. In addition to providing CBPR with new options for data collection, low-cost sensors allow researchers and communities to examine high time-resolution data alongside community-member knowledge, which offers yet another way to engage community expertise to better understand the potential impact of local emission sources, such as oil and gas operations. Through this mixed methods approach, we report on how research engages community expertise as central to the research process and extends knowledge on local health impacts from oil development. We also suggest how community residents living nearby urban oil drilling facilities can contribute to locally relevant health interventions and lend their knowledge to support the development of relevant policy measures. 

## 2. Methods

### 2.1. Neighborhood Profile

The West Adams and University Park neighborhoods in South Los Angeles host well established well fields with sustained and active oil development. CalEnviroscreen identifies these neighborhoods as having significant residential vulnerability when compared to all census tracts Statewide. Esperanza Community Housing and Redeemer Community Partnership are member organizations of STAND LA and the two organizations that took the community lead on this study as the sites are in their service provision area. Esperanza is a longstanding community organization in the University Park area, where the AllenCo oil site is located. The neighborhood is predominantly Latino (76%) with 72% of residents living 200% below the poverty line and 81% renters. Redeemer Community Partnership is a community development corporation in the West Adams neighborhood for over 25 years and has been organizing the community around the Jefferson Drill Site. The neighborhood is 87% residents of color, with 58% Latino and 20% African-American. 20% of the population is under the age of 5 (as compared to 7% for Los Angeles County), 68% of residents live 200% below the poverty line, and 69% of residents are renters (Table 2). 

### 2.2. Study Area and Sample Selection 

**Figure 1:**
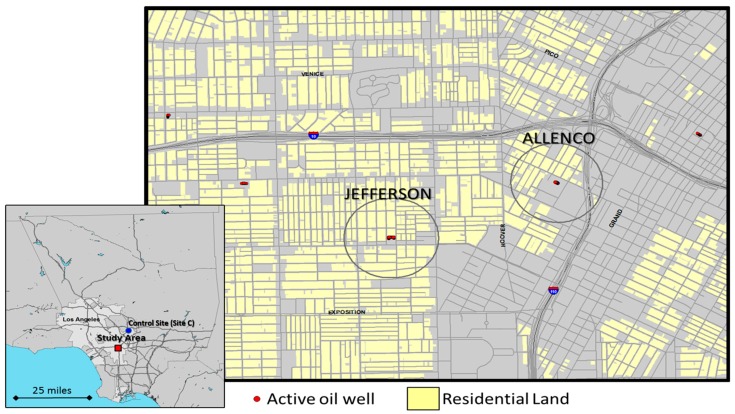
Location map. Study area is located in the mid-city area of Los Angeles, just west of downtown. Circles are 1500 ft. radius buffers surrounding active wells. Note active oil wells in other nearby residential neighborhoods.

The study areas were defined to represent the neighborhoods surrounding the wells and production facilities at two locations that produce oil from the Las Cienegas oilfield (Figure 1). Study areas were defined by constructing a circular buffer using ArcGIS (Esri, Redlands, CA, USA), with a 1500 ft. radius surrounding the outer perimeter of the two oil production sites (Figure 1). The Jefferson Drill Site (Jefferson) is located in the West Adams neighborhood, and the AllenCo Drill Site (AllenCo) is located in the University Park neighborhood of Los Angeles. We chose a 1500 ft. buffer based on distances considered by other urban cities, such as Dallas [35]. It should be noted that Site C was utilized only for the air monitoring portion of the study; its description appears subsequently. 

These buffers were then used to capture census polygons, sensitive land uses, land use information, and residential street addresses used in the study. From the residences inside each buffer, a random sample of street addresses was selected from tax parcel records obtained from the LA County Office of the Assessor, for the purposes of administering the survey. To determine the minimum number of households to survey at each study site, we calculated the sample size required from each population to estimate the value of a continuous variable with a 95% CI and a 10% margin of error. The number of random sample addresses was determined using an estimate of the variability in responses expected at a 95% CI. We also performed a one-sample proportion test for physician-diagnosed asthma (age 0–17) and emergency room visits by asthmatics to determine the minimum proportions in our surveyed population that we could detect as being significantly different from the overall rates measured for LA County by CHIS and the LA County Health Department for a range of possible sample sizes. Based on this analysis, we selected a target minimum sample size of 76 households around AllenCo and 84 households around Jefferson, conducting the surveys at the addresses identified using a random sampling algorithm to ensure systematic sample coverage (Human Subjects Code: Sham-F16006).

### 2.3. Survey Instrument

We designed a survey instrument in collaboration with Esperanza Community Housing and Redeemer Community Partnership to identify baseline informational questions that were of community importance, such as ratings of the environmental quality of their neighborhood, feelings of safety living in the neighborhood, and resident knowledge of the site. For the health portion of the survey, community residents were primarily interested in respiratory health and reproductive health, in particular asthma and birth outcomes. We drew from questions asked in the California Health Interview Survey (CHIS) for which the questionnaires are publicly available, in order to compare our study area to the CHIS survey data. CHIS is a random-dial telephone survey that is conducted on a continuous basis allowing the survey to generate timely one-year estimates. The survey provides representative data on all California counties and over samples and creates small-area estimates in Los Angeles County. Surveys were created in Qualtrics, and paper copies were generated for field use. 

### 2.4. CBPR Method for Administration of Resident Surveys: Data Collection and Community Building

Health surveys are a well-recognized method of community organizing in an environmental justice context [36]. The survey provided a vehicle for community organizing and education about issues of concern in the neighborhood. Residents were able to provide their information if they wanted to be contacted for report back on the survey or other community events. In partnership with Esperanza’s trained network of community health workers, *Promotoras de Salud* in Action, we conducted the door-to-door surveys of residents in Spanish and English. *Promotora de Salud* networks are recognized within CBPR environmental justice research [37]. *Promotoras de Salud* live and work in the community, are engaged long-term in community building, and have a baseline of trust in the neighborhood. They are agile at accessing residents, many of whom work in service sector jobs, the night shift, or have other non-traditional working hours. We also trained four bilingual Occidental College students to conduct surveys alongside *promotoras*. Using the addresses generated from the random household sample, we visited each household on our list starting in March 2016 and continued through May 2016. If we could not find anyone at home, we returned on different days and at different times until the survey could be completed. In addition to our random household sample, residents became interested in the survey as they saw surveyors in conversation with other residents. These residents were included in a snowball sample collected to supplement the randomly designed surveys if the address fell within the 1500 ft. buffer. Through these methods, we were able to achieve a high survey response rate. 

In addition to asthma and respiratory health, we asked questions about infertility and birth outcomes because community organizers were interested in these variables. We provided this self-reported information back to the community but we do not include an analysis of these data here as we did not have specific enough data to do a birth outcomes analysis, and had informed residents in advance. Thus, that information serves to inform internal community efforts moving forward and should be considered for future research. 

### 2.5. Survey Analysis

Surveys were coded to correspond with a created codebook and entered by hand into Qualtrics. All subsequent analyses were performed in the statistical software package R (version 3.3.0; R Development Core Team, 2016). We calculated proportions for demographic variables and health conditions of interest, excluding respondents/households that did not answer the corresponding question. 

#### 2.5.1. Health Insurance

We collected data on the type of health insurance for all members of surveyed households. To determine the proportion of people in surveyed households that were uninsured, we subtracted the total number of people with each type of health insurance from the total number of residents of households surveyed. Individuals that responded “unknown” for health insurance type were excluded from the analysis.

#### 2.5.2. Asthma and Asthmatic Hospitalization Rate

To account for age-dependent differences in asthma diagnoses, we calculated an age-adjusted asthma rate. We determined the rates of diagnosed asthma for survey respondents in two age categories (18–64 years and 65+ years) and for children (0–17 years) residing in surveyed households. Households with smokers were excluded from the analysis. To estimate the age-adjusted asthma rate, the asthma rate for each age category (0–17, 18–64 and 65+) was weighted using the age distribution for Los Angeles County. The asthma hospitalization rate was calculated only for respondents that had previously been diagnosed with asthma. 

We compared self-reported demographic and health outcomes from this survey to publicly available data for both Service Planning Area 6 (SPA6) and Los Angeles County from the California Health Interview Survey (CHIS) and the American Community Survey 5-year rollup 2009–2013 [38]. To determine if self-reported proportions differed from previously published proportions for Los Angeles County and SPA6, we performed one-proportion z-tests. 

### 2.6. Air Quality Monitoring with Low Cost Sensors

Low-cost sensor systems are typically small and low power, which makes them fairly easy to deploy at potential sites within the community (e.g., homes, schools, or businesses). This flexibility allows researchers and community members to work together in choosing sites that will best inform the research question. In this pilot study, the monitors were set-up and maintained by community-based research partners at three field sites. These sites include three residences—one near an active drill site (West Adams, labeled Site A), one across the street from an inactive drill site (AllenCo, labeled Site B), and one in an area with no drilling (our control site, labeled Site C). Sites A and B were located in the Study Area (Figure 1), whereas Site C was located roughly seven miles away in Northeast Los Angeles. Site C was intended to serve as a comparison for the low-cost sensor data portion of the study, and no other measures were taken at this location. All sites were relatively similar in terms of land use and proximity to other major pollution sources (e.g., highways). The sites were selected to provide a preliminary example of what sensors can tell us regarding the differences in methane levels/trends in areas with drilling versus those without drilling. Issues with the data logging system at Site B resulted in data loss, and this incomplete data was not included in the analysis of the field deployment data—meaning the figures below illustrate the comparison between the West Adams site and the control site. The system used for data logging and acquisition is an open-source design termed a U-pod [39]. This system supports several air quality and environmental sensors including the Figaro TGS 2600 metal oxide-semiconductor VOC sensor and the RHT03 temperature and relative humidity sensor. Field calibration for methane was used to convert raw sensor signal data to volumetric concentrations; this method involves co-locating the low-cost sensors with a high-quality, calibrated reference instrument and then using regression analysis to develop a predictive model [40]. In this case, the U-pods were co-located together with a Baseline-Mocon Series 9000 NMHC Analyzer before and after the field deployment, and data from the monitor with the most complete set of calibration data were used to generate the predictive model that was applied to the normalized data from the remaining monitors. Data used for calibration and field data analysis were minute-averaged. It should be noted that these results are preliminary and intended to explore the potential for this technology in the context of CBPR; more rigorous calibration and validation of these sensors is explored in a separate paper [41].

## 3. Results

### 3.1. Demographics

We surveyed 84 households comprising 315 residents in University Park and 119 households comprising 498 residents in West Adams. In both sites, more than 50% of surveyed households had incomes of $20,000 or less (University Park: 57.1%, West Adams: 53.7%). Median household income according to census data (2010–2015 ACS 5-year rollup) for West Adams is $25,980, and for University Park is $20,115. For both sites, the proportions of households with self-reported incomes of less than $20,000 were significantly higher than for Los Angeles County (LA County: 17.2%; University Park: 57.1% [95% CI: 48.3–65.9%], *n* = 70, z = 8.85, *p* < 0.001; West Adams: 53.6% [95% CI: 45.5–61.8%], *n* = 82, z = 8.75, *p* < 0.001) and SPA6 (SPA6: 30.1%; University Park: 57.1% [95% CI: 46.4–67.9%], *n* = 70, z = 4.93, *p* < 0.001, West Adams: 53.6% [95% CI: 43.7–63.6%], *n* = 82, z = 4.65, *p* < 0.001). For both sites, the reported proportions of residents on Medi-Cal were significantly higher than the previously reported rate for Los Angeles County (LA County: 30.1%; University Park: 46.0% [95% CI: 40.9–51.1%], *n* = 315, z = 6.21, *p* < 0.001; West Adams: 44.8% [95% CI: 40.8–48.9%], *n* = 484, z = 6.21, *p* < 0.001 ) but not the reported rate for SPA6 (SPA6: 50.1%; University Park: 46.0% [95% CI: 40.5–51.6%], *n* = 315, z = −1.44, *p* = 0.925, West Adams: 44.8% [95% CI: 40.4–49.3%], *n* = 484, z = −2.31, *p* = 0.989). In University Park, the proportion of uninsured residents was significantly higher than the previously reported rate of 10.4% for Los Angeles County (16.2% [95% CI: 12.6–19.7%], *n* = 315, z = 2.29, *p* = 0.01) and 12.0% for SPA6 (16.2% [95% CI: 12.6–19.8%], *n* = 315, z = 2.29, *p* = 0.01). In West Adams, the proportion of uninsured residents was not significantly different from Los Angeles County (11.0% [95% CI: 8.2–13.7%], *n* = 484, z = 0.40, *p* = 0.35) or SPA6 (11.0% [95% CI: 8.1–13.8%], *n* = 484, z = −0.71, *p* = 0.76) rates [42]. 

### 3.2. Community Knowledge and Experiences 

Many respondents (University Park: 45.8%, West Adams: 38.9%) reported that they did not have prior knowledge of the oil production site. Those that knew of the facility (University Park: 33%, West Adams: 42.4%) had questions about the site. Most respondents (University Park: 78.5%, West Adams: 76.9%) answered they “definitely did not” or “probably did not” know how to make a report to the South Coast Air Quality Management District or other agency. Some respondents (University Park: 15.7%, West Adams: 27.5%) reported that odors from the site had prevented daily activities. Very few of the respondents had previously reported odors to the gas company (University Park: 2.4%, West Adams: 3.6%), the Los Angeles Department of Public Health (University Park: 2.4%, West Adams: 1.8%), the South Coast Air Quality Management District (University Park: 2.4%, West Adams: 2.7%), or any other entity (University Park: 3.7%, West Adams: 1.8%). 

### 3.3. Reported Health Symptoms

For both University Park and West Adams survey sites, the age-adjusted rate of diagnosed asthma was significantly higher than in SPA6 (SPA6: 9.8%; University Park: 16.1% [95% CI: 8.6–23.6%], z = 3.41, *p* < 0.001; West Adams: 20.3% [95% CI: 13.5–27%], z = 3.02, *p* = 0.001). Asthma prevalence in West Adams but not University Park was significantly higher than that in Los Angeles County (LA County: 13.0%; University Park: z = 0.82, *p* = 0.21; West Adams: z = 2.10, *p* = 0.02) [43]. Moreover, 15.5% [95% CI: 8.5–22.5%] of all respondents in West Adams and 12.1% [95% CI: 4.7–19.6%] of all respondents in University Park reported experiencing asthma symptoms (coughing and wheezing) on a weekly or daily basis. Respondents with diagnosed asthma reported rates of ER visits in the previous 12 months that were not significantly higher than SPA6 (SPA6: 26.8%; University Park: 18.8% [95% CI: −3.0–40.5%], *n* = 16, z = −0.73, *p* = 0.766; West Adams (25.0% [95% CI: 8.59–41.4%], *n* = 28, z = −0.21, *p* = 0.585) or Los Angeles County (LA County: 17.0%; University Park: 18.8% [95% CI: −3.0–40.5%], *n* = 16, z = 0.19, *p* = 0.426; West Adams: (25.0% [95% CI: 8.59–41.4%], *n* = 28, z = 1.13, *p* = 0.129) [44]. 

### 3.4. Low-Cost Sensor Calibration and Field Data

Comparing the calibrated sensor data and the methane reference data reveals an R-squared of 0.45 and a root mean squared error (RMSE) of 0.14 ppm. While the R-squared seems low, given the RMSE and the range of methane values observed during calibration, the signal-to-noise ratio is approximately 4.79, indicating that enhancements in methane above our error are likely visible. Additionally, researchers in a study in rural Alaska noted that, despite low R-squared values when comparing calibrated sensor data to validation data, the methane’s diurnal patterns were clearly resolved, meaning the sensor data still included valuable information [34]. When we compare the low-cost sensors’ performance, there is an average correlation coefficient of 0.90 for each sensor pairs’ calibrated data, which demonstrates that the sensors are well correlated with each other when co-located and the calibration model is performing consistently.

**Figure 2:**
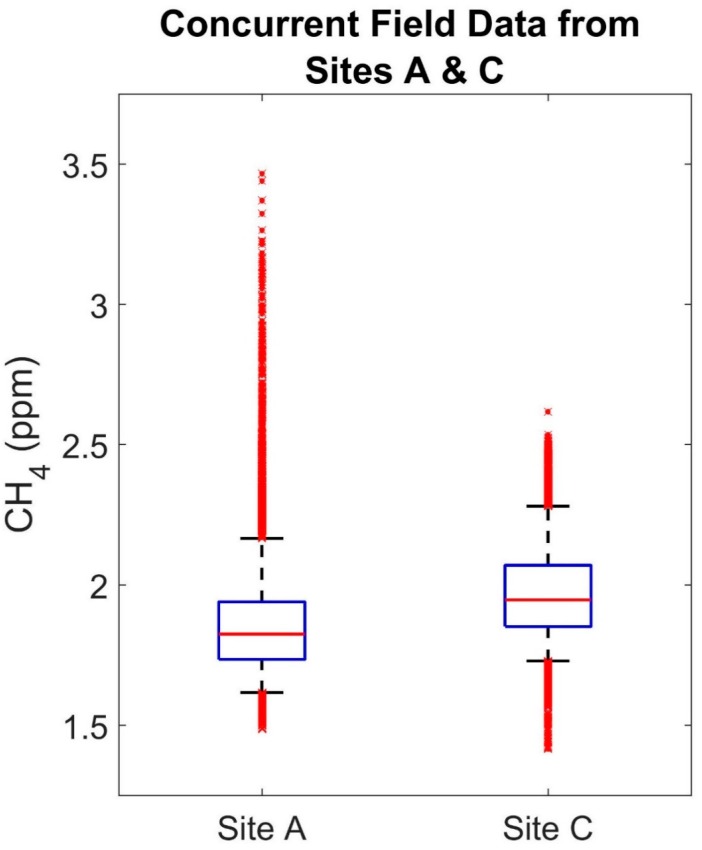
Concurrent field data from Sites A and C.

**Figure 3:**
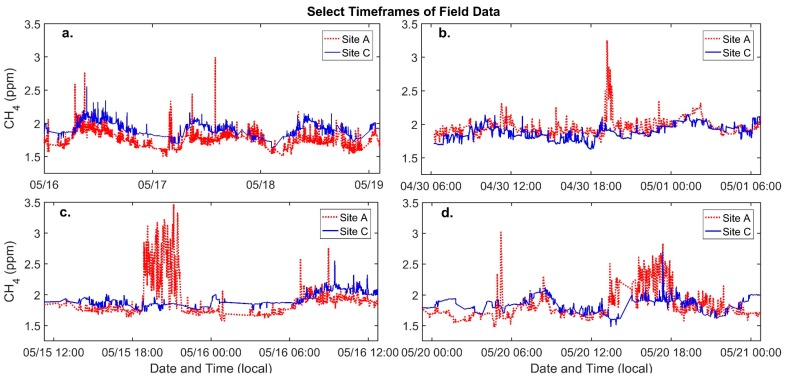
Select timeframes of field data that illustrate relatively large differences in methane between Sites A and C. (**a**) a period of four days including several examples of enhanced methane; (**b**) a 24-h period including an approximately 1 ppm increase in methane; (**c**) a 24-h period depicting an approximately three-hour long methane enhancement; (**d**) a 24-h period including several enhancements in methane.

The correlation coefficient for the sensors at Sites A and C decreases to 0.35 for the field data indicating differences between these two sites that are visible in the sensor data. Figure 2 compares the complete calibrated field data from Sites A and C. While the median is slightly higher at Site C, most of the data from both sites is varying within a similar range as is demonstrated by similarity in difference between the 95th and 5th percentiles for Sites A and C (0.548 and 0.551 ppm, respectively). However, an important difference between the two sites is the presence of short-term increases in methane occurring at one site and not the other, which are larger and more frequent at Site A (nearly 1 ppm larger). Figure 3b–d illustrate some of these increases, confirming that, overall, the diurnal trends and methane ranges at each site are similar and that these short-term events may be driving the difference in correlation. 

## 4. Discussion

Our random household sample and exposure monitoring within 1500 ft. of oil development sites is the first study in partnership with residents living in very close proximity to oil development in Los Angeles. It is also the first study, to our knowledge, to compare the self-reported health of residents within 1500 ft. of oil development to residents in the broader area of South Los Angeles (SPA6) and Los Angeles County. The testing of low-cost sensors supports community organization efforts to collect air monitoring data as related to oil development in their neighborhoods. There are currently no buffers or setbacks between sensitive land uses, such as homes and schools, and oil development in Los Angeles. West Adams and University Park are located within an area identified by the State of California as having high vulnerability to the health impacts of pollution due to the SES status of residents exposed to multiple sources of pollution. More than 50% of residents reported household incomes of $20,000 or less as compared to 29% of households in SPA6 and 17% in LA County. The median household income is lower in both communities than in the City (Table 2). Within the West Adams buffer, 20% of residents identify as Non-Hispanic Black as compared to 10% in the City. 76% of residents within the University Park buffer and 58% in West Adams buffer identify as Hispanic as compared to 50% in the City. 

We gathered community experiences living adjacent to oil development sites. Many residents (University Park: 45.8%, West Adams: 38.9%) living within 1500 ft. did not know that an oil development site was located in the neighborhood. This may be due to tall walls and landscaping surrounding both sites, and to visible private property and no trespassing signage. From our survey, one of the main burdens appeared to be odors, which some respondents reported prevented daily activities (University Park: 15.7%, West Adams: 27.5%). However, only a few respondents said they had reported odors or any health symptoms to the gas company, the Los Angeles Department of Public Health, the South Coast Air Quality Management District, or any other entity, as most responded that they lacked information as to how to report. Further, since most residents are unaware of these activities, they may attribute symptoms to allergies or general poor air quality. 

Oil and gas development is associated with degraded air quality and exposure to air pollution [45,46] as well as exacerbated respiratory conditions and asthma [47]. For both University Park and West Adams, compared with SPA6, resident-reported asthma prevalence was significantly higher. Respondents in West Adams (15.5%) and University Park (12.1%) reported experiencing asthma symptoms of coughing and wheezing on a weekly or daily basis. Decreases in ambient pollution levels in Southern California have been associated with statistically significant decreases in asthma-related symptoms in children [48]. Children under the age of 5 living within the West Adams buffer area represent 20% of the population as compared to 7% of residents in the city of Los Angeles, and this group is more biologically sensitive to air pollution health impacts. Future studies might consider children’s health specifically. Respondents with diagnosed asthma in our sample did not report higher rates of emergency room visits in the previous 12 months (West Adams 25%; University Park 19%) than previously reported in SPA6 or LA County, but our sample size was small (16 diagnosed asthmatics for UP (University Park) and 28 diagnosed asthmatics for WA (West Adams)). 

The reliance on self-reported information is a possible limitation of the study. Surveys are not an optimal method for estimates for some kinds of health outcomes of interest, such as birth defects, especially in a population that may be transient. However, the use of questions from a validated survey facilitated comparison for asthma and respiratory health. While both Jefferson and AllenCo have long operated in these neighborhoods, AllenCo was closed at the time of the survey to address their lack of emissions controls. We report both University Park and West Adams rates here given the significant community interest in both fields and concern over AllenCo’s reopening. Another limitation of the survey could be awareness bias. The study questionnaire asked respondents about knowledge of their environment and the oil development, as well as health status. One way to reduce this bias may be to control for the effect of awareness [6]. In our case, community organizations were interested in learning both about environmental awareness and self-reported health. 

### Air Quality Monitoring

The similarities between the data from Sites A and C seen in the diurnal patterns and the ranges of methane variation are likely explained by regional methane trends and meteorology. There was an offset between the two datasets (seen in the differing medians); however, this offset was less than the RMSE determined during calibration. Spatial differences occurred at a finer temporal scale as can be observed in Figure 3, which depicts periods of elevated methane lasting from approximately 10 min to up to 3 h. These events included differences in methane between the two sites greater than 1.0 ppm, well above the RMSE of the calibration model (0.14). Given that these events occurred at one site and not the other, they were likely the result of an emission source nearer to Site A. This was even more evident for the events that occurred during daytime hours when more, atmospheric mixing is typically taking place [49]. Additional measurements would aid in further narrowing down the source of these events. For example, wind speed and direction information combined with data from multiple sensors might point to the origin of emissions. While data from different types of low-cost sensors could possibly provide more information on the composition of the plumes causing these events. Another benefit of utilizing low-cost sensors in a CBPR context is that local experience, such as observations about local activities or odors, can improve interpretations of the data. On one day depicted in Figure 3, nearby residents reported seeing heavy equipment being used at the active drill site. If similar methane enhancements were observed every time this activity occurred, it would indicate a correlation worthy of further investigation. Examining this qualitative data alongside quantitative data provided by low-cost sensors may result in a more comprehensive understanding of the community’s experiences, which could in turn carry through and inform community-based action or potentially policy recommendations.

## 5. Conclusions

Urban oil development is an under-researched issue. It is difficult to examine oil-development-related impacts in a cumulatively burdened context, but it is critical to do so. Our preliminary community-based survey and low-cost sensor field experiment considers resident health and the rights of residents to have knowledge about their communities and supports hypothesis generation for future air monitoring or health studies. It also points to the need for regulatory agencies to provide community education about reporting experiences such as odors as well as facilitate diverse methods to be able to do so. It leads to questions that require more complex scientific design than possible in this study with limited resources and raises the imperative that communities be involved in the research. Studies on oil and gas development have well associated distance with worsened air pollution, an issue of significant concern in Los Angeles. The Inter-Environmental Health Sciences Core Center Working Group recommends that ambient air quality be measured at active drilling sites and be compared with baseline measurements in adjacent areas without active oil and gas drilling [2]. A low-cost sensor approach to measure methane and total non-methane hydrocarbons could facilitate these types of comparisons, providing preliminary information on potential exposure differences that could inform additional air quality sampling or possibly action by residents or policymakers to lower exposures. This recommendation, in particular, is relevant to Los Angeles oil development and should be considered in future studies. One possible avenue is to employ a daily log model where participants note their symptoms and experiences over a period of time while also collecting local sensor data. These could then be examined side by side. Buffers/setbacks are well recognized as protective by the regulatory authorities such as local air districts and should be incorporated into neighborhood oil development sites to protect community health [50]. This research supports community-based research methods in the context of oil and gas development. CBPR-generated data can support community building as residents seek a setback or buffer between sensitive land uses and oil development citywide.

## Figures and Tables

**Table 1:** Sensitive land uses in selected areas hosting oil production facilities.

Location	Number of Schools	Childcare Facilities	Schools per 10,000 People	Childcare per 10,000 People	Childcare per sq. Mile
L.A. County	3036	3903	3.09	3.98	1.6
L.A. City	1087	1385	2.88	3.67	2.9
Within 1500 ft. of an active L.A. City Well	40	29	3.25	2.35	1.5
University Park: AllenCo	5	2	7.83	3.13	8.0
Historic West Adams: Jefferson	1	2	1.29	2.59	8.0
Historic West Adams: Murphy	3	1	5.44	1.81	4.0
Wilmington: Warren E&P	0	1	0.00	2.35	2.4
Baldwin Hills: Inglewood Oil Field	2	7	3.64	2.35	4.4

**Table 2:** Demographics of West Adams and University Park within the 1500 ft. buffer.

Population	West Adams Buffer Area 1500 ft. around Jefferson Oil Field	University Park Buffer Area, 1500 ft. around AllenCo Oil Field	City of Los Angeles
Total Population	6641	5401	2,546,606
% Age 5 or under	20.90%	5.31%	7.63%
% Age 65 or older	9.92%	6.94%	6.95%
% People of Color	87.82%	84.17%	72.85%
% Non-Hispanic Black	20.86%	8.17%	9.99%
% Non-Hispanic White	12.18%	15.83%	27.15%
% Hispanic	58.20%	76.00%	50.85%
% Linguistically Isolated	23.42%	39.21%	12.37%
% Less Than High School	42.49%	46.72%	18.91%
Per Capita Income	$11,194	$11,203	$18,839
Median Household Income	$23,912	$20,115	$37,723
Poverty (LT 150%)	51.51%	59.39%	20.57%
Poverty (LT 200%)	64.88%	72.30%	27.57%
% Renters	68.77%	81.13%	34.70%
Median Household Size	2.7	3.4	1.7

## References

[1] Shonkoff S.B., Hays J., Finkel M.L. (2014). Environmental public health dimensions of shale and tight gas development. Environ. Health Perspect..

[2] Penning T.M., Breysse P.N., Gray K., Howarth M., Yan B. (2014). Environmental health research recommendations from the inter-environmental health sciences core center working group on unconventional natural gas drilling operations. Environ. Health Perspect..

[3] Balazs C.L., Morello-Frosch R. (2013). The three R’s: How community-based participatory research strengthens the rigor, relevance, and reach of science. Environ. Justice.

[4] Meng Q., Ashby S. (2014). Distance: A critical aspect for environmental impact assessment of hydraulic fracking. Extr. Ind. Soc..

[5] McKenzie L.M., Guo R., Witter R.Z., Savitz D.A., Newman L.S., Adgate J.L. (2014). Birth outcomes and maternal residential proximity to natural gas development in Rural Colorado. Environ. Health Perspect..

[6] Rabinowitz P.M., Slizovskiy I.B., Lamers V., Trufan S.J., Holford T.R., Dziura J.D., Peduzzi P.N., Kane M.J., Reif J.S., Weiss T.R. (2015). Proximity to natural gas wells and reported health status: Results of a household survey in Washington County, Pennsylvania. Environ. Health Perspect..

[7] Chilingar G.V., Endres B. (2005). Environmental hazards posed by the Los Angeles Basin Urban Oilfields: An historical perspective of lessons learned. Environ. Geol..

[8] Division of Oil, Gas & Geothermal Resources. (DOGGR) Well Finder. http://www.conservation.ca.gov/dog.

[9] Sadd J., Shamasunder B. (2015). Oil Extraction in Los Angeles: Health, Land Use, and Environmental Justice Consequence. Drilling Down: The Community Consequences of Expanded Oil Development in Los Angeles.

[10] Quam-Wickham N. (1998). “Cities sacrificed on the altar of oil”: Popular opposition to oil development, in 1920s Los Angeles. Environ. Hist..

[11] Community & Tribal Programs Group & Ambient Air Monitoring Group (2007). Technical Guidance for the Development of Tribal Air Monitoring Programs.

[12] Morello-Frosch R., Shenassa E.D. (2006). The environmental “riskscape” and social inequality: Implications for explaining maternal and child health disparities. Environ. Health Perspect..

[13] Morello-Frosch R., Pastor M., Sadd J. (2001). Environmental justice and Southern California’s “Riskscape” the distribution of air toxics exposures and health risks among diverse communities. Urban Aff. Rev..

[14] Office of Environmental Health Hazard Assessment (OEHHA) CalEnviroScreen 3.0. https://oehha.ca.gov/calenviroscreen/report/calenviroscreen-30.

[15] California Environmental Protection Agency (2017). Designation of Disadvantaged Communities Pursuant to Senate Bill 535 (De Leon).

[16] Cole L.W., Foster S. (2001). From the Ground up: Environmental Racism and the Rise of the Environmental Justice Movement.

[17] Brown P. (1992). Popular epidemiology and toxic waste contamination: Lay and professional ways of knowing. J. Health Soc. Behav..

[18] Corburn J. (2002). Combining community-based research and local knowledge to confront asthma and subsistence-fishing hazards in Greenpoint/Williamsburg, Brooklyn, New York. Environ. Health Perspect..

[19] Witter R.Z., McKenzie L., Stinson K.E., Scott K., Newman L.S., Adgate J. (2013). The use of health impact assessment for a community undergoing natural gas development. Am. J. Public Health.

[20] Macey G.P., Breech R., Chernaik M., Cox C., Larson D., Thomas D., Carpenter D.O. (2014). Air concentrations of volatile compounds near oil and gas production: A community-based exploratory study. Environ. Health.

[21] Abdullah K., Malloy T., Stenstrom M.K., Suffet I.H. (2017). (Mel) Toxicity of acidization fluids used in California oil exploration. Toxicol. Environ. Chem..

[22] Stringfellow W.T., Camarillo M.K., Domen J.K., Sandelin W.L., Varadharajan C., Jordan P.D., Reagan M.T., Cooley H., Heberger M.G., Birkholzer J.T. (2017). Identifying chemicals of concern in hydraulic fracturing fluids used for oil production. Environ. Pollut..

[23] Sahagun L. (2013). Chemical odor, kids’ nosebleeds, few answers in South L.A. Neighborhood. Los Angeles Times.

[24] Haley M., McCawley M., Epstein A.C., Arrington B., Bjerke E.F. (2016). Adequacy of current state setbacks for directional high-volume hydraulic fracturing in the Marcellus, Barnett, and Niobrara Shale Plays. Environ. Health Perspect..

[25] Fry M. (2013). Urban gas drilling and distance ordinances in the Texas Barnett Shale. Energy Policy.

[26] Guerin E. (2017). LA to Study Banning Oil Production around Homes, Schools, Hospitals and Other Public Places. http://www.scpr.org/news/2017/04/19/70946/la-to-study-banning-oil-production-around-homes-sc/.

[27] California Air Resources Board (2005). Air Quality and Land Use Handbook: A Community Health Perspective.

[28] Lewis A.C., Lee J.D., Edwards P.M., Shaw M.D., Evans M.J., Moller S.J., Smith K.R., Buckley J.W., Ellis M., Gillot S.R. (2016). Evaluating the performance of low cost chemical sensors for air pollution research. Faraday Discuss.

[29] Snyder E.G., Watkins T.H., Solomon P.A., Thoma E.D., Williams R.W., Hagler G.S.W., Shelow D., Hindin D.A., Kilaru V.J., Preuss P.W. (2013). The changing paradigm of air pollution monitoring. Environ. Sci. Technol..

[30] Masson N., Piedrahita R., Hannigan M. (2015). Approach for quantification of metal oxide type semiconductor gas sensors used for ambient air quality monitoring. Sens. Actuators B Chem..

[31] Jovašević-Stojanović M., Bartonova A., Topalović D., Lazović I., Pokrić B., Ristovski Z. (2015). On the use of small and cheaper sensors and devices for indicative citizen-based monitoring of respirable particulate matter. Environ. Pollut..

[32] Piedrahita R., Xiang Y., Masson N., Ortega J., Collier A., Jiang Y., Li K., Dick R.P., Lv Q., Hannigan M. (2014). The next generation of low-cost personal air quality sensors for quantitative exposure monitoring. Atmos. Meas. Tech..

[33] Mead M.I., Popoola O.A.M., Stewart G.B., Landshoff P., Calleja M., Hayes M., Baldovi J.J., McLeod M.W., Hodgson T.F., Dicks J. (2013). The use of electrochemical sensors for monitoring urban air quality in low-cost, high-density networks. Atmos. Environ..

[34] Eugster W., Kling G.W. (2012). Performance of a low-cost methane sensor for ambient concentration measurements in preliminary studies. Atmos. Meas. Tech..

[35] Loftis R.L. (2013). Dallas OKs Gas Drilling Rules That Are among Nation’s Tightest. Dallas Morning News.

[36] Cohen A.K., Lopez A., Malloy N., Morello-Frosch R. (2016). Surveying for environmental health justice: Community organizing applications of community-based participatory research. Environ. Justice.

[37] Minkler M., Garcia A.P., Williams J., LoPresti T., Lilly J. (2010). Sí Se Puede: Using participatory research to promote environmental justice in a Latino Community in San Diego, California. J. Urban Health.

[38] U.S. Census Bureau (2014). American Community Survey 5-Year Dataset 2009–2013.

[39] Masson N., Piedrahita R., Hannigan M. (2015). Quantification method for electrolytic sensors in long-term monitoring of ambient air quality. Sensors.

[40] Spinelle L., Gerboles M., Villani M.G., Aleixandre M., Bonavitacola F. (2015). Field calibration of a cluster of low-cost available sensors for air quality monitoring. Part A: Ozone and nitrogen dioxide. Sens. Actuators B Chem..

[41] Collier-Oxandale A., Hannigan M., Casey J.G., Piedrahita R., Johnston J. (2018). Assessing a low-cost methane sensor quantification system for use in complex rural and urban environments. Atmos. Meas. Tech..

[42] UCLA Center for Health Policy Research Type of Current Health Insurance Coverage (Los Angeles, SPA South). http://ask.chis.ucla.edu.

[43] UCLA Center for Health Policy Research AskCHIS 2015. Ever Diagnosed with Asthma (Los Angeles, SPA South). http://ask.chis.ucla.edu.

[44] UCLA Center for Health Policy Research AskCHIS 2015. Had Emergency Room/Urgent Care Visit for Asthma within Past 12 Months (Current Asthmatics) (Los Angeles, SPA South). http://ask.chis.ucla.edu.

[45] Thompson T.M., Shepherd D., Stacy A., Barna M.G., Schichtel B.A. (2017). Modeling to evaluate contribution of oil and gas emissions to air pollution. J. Air Waste Manag. Assoc..

[46] Webb E., Hays J., Dyrszka L., Rodriguez B., Cox C., Huffling K., Bushkin-Bedient S. (2016). Potential hazards of air pollutant emissions from unconventional oil and natural gas operations on the respiratory health of children and infants. Rev. Environ. Health.

[47] Rasmussen S.G., Ogburn E.L., McCormack M., Casey J.A., Bandeen-Roche K., Mercer D.G., Schwartz B.S. (2016). Association between unconventional natural gas development in the Marcellus Shale and Asthma Exacerbations. JAMA Intern. Med..

[48] Berhane K., Chang C.-C., McConnell R., Gauderman W.J., Avol E., Rapapport E., Urman R., Lurmann F., Gilliland F. (2016). Association of changes in air quality with bronchitic symptoms in children in California, 1993–2012. JAMA.

[49] Bamberger I., Stieger J., Buchmann N., Eugster W. (2014). Spatial variability of methane: Attributing atmospheric concentrations to emissions. Environ. Pollut..

[50] Sadd J.L., Pastor M., Morello-Frosch R., Scoggins J., Jesdale B. (2011). Playing it safe: Assessing cumulative impact and social vulnerability through an environmental justice screening method in the South Coast Air Basin, California. Int. J. Environ. Res. Public Health.

